# Low-Dose Sodium Salicylate Promotes Ovulation by Regulating Steroids via CYP17A1

**DOI:** 10.3390/ijms24032579

**Published:** 2023-01-30

**Authors:** Tao Li, Xuehua Ren, Tianjiao Li, Lian Yu, Mingming Teng, Yi Zheng, Anmin Lei

**Affiliations:** 1 Shaanxi Stem Cell Engineering and Technology Center, College of Veterinary Medicine, Northwest A&F University, Yangling, Xianyang 712100, China; 2 Key Laboratory for Animal Genetics, Breeding and Reproduction of Shaanxi Province, College of Animal Science and Technology, Northwest A&F University, Yangling, Xianyang 712100, China

**Keywords:** superovulation, sodium salicylate, CYP17A1, ovary

## Abstract

To meet the current demand of assisted reproduction and animal breeding via superovulation and reduce the impact of hormone drugs, it is necessary to develop new superovulation drugs. This study examined the role of inflammation and steroids in ovulation. Sodium salicylate can regulate inflammation and steroids. However, the effect of sodium salicylate on ovulation has not been studied. In this study, mice were intraperitoneally injected with different concentrations of sodium salicylate for four consecutive days. The effects of sodium salicylate on oocyte quality and on the number of ovulations were examined, and these effects were compared with those of pregnant horse serum gonadotropin (PMSG)/follicle-stimulating hormone (FSH) treatment. We found that low-dose sodium salicylate increased the levels of ovulation hormones and inflammation by promoting the expression of CYP17A1. Sodium salicylate had the same effect as the commonly used superovulation drug PMSG/FSH and reduced the histone methylation level. Sodium salicylate can promote ovulation in mice and Awang sheep. It can greatly decrease the use of hormone drugs, reduce breeding costs and physical impacts, and can thus be used for livestock breeding.

## 1. Introduction

Superovulation is a technique that promotes multiple follicle development in the ovary through the injection of exogenous hormones into females, which can maximize the number of ovulations. This strategy can be used clinically in humans for assisted reproductive technology, embryo recovery and the transfer of single-birth livestock, and the breeding of multiple-birth livestock for expansion [1]. There are many types of hormones currently being used for superovulation in animals, but the most commonly used hormones are roughly divided into two categories. One category is pregnant horse serum gonadotropin (PMSG) and follicle-stimulating hormone (FSH), which promote oocyte development; the other category is human chorionic gonadotropin (hCG) and luteinizing hormone (LH) [2], which promote ovulation. Among them, the combination of PMSG and HCG is commonly used for animal superovulation [3]. Superovulation is mainly affected by factors, such as hormone type, dose, donor species, and donor age. As one of the key technologies for embryo transfer, it has been fully promoted and used in mice, cattle, sheep, and humans, and the combination of embryo transfer technology has greatly improved reproductive potential and reproductive efficiency [4]. The frequent use of superovulation hormones typically leads to a decline in the quality of some follicles and oocytes [5,6,7], which has an impact on later embryo implantation and pregnancy [8,9]. Therefore, it is necessary to find new superovulation drugs to alleviate this outcome, enrich the selection of superovulation drugs, and reduce the use of hormone drugs and the generation of drug resistance.

Ovulation and inflammation are similar in many ways. The LH surge regulates the production of steroids, prostaglandins (PG), chemokines, and cytokines by granulosa and theca cells during ovulation. Conversely, these factors can act as inflammatory mediators to activate nonimmune ovarian cells and resident immune cells within the ovary [10]. 

Ovulation occurs through the joint actions of various hormones secreted by the hypothalamus–pituitary gland. The LH surge can promote the orderly process of ovulation by activating the mitogen-activated protein kinase (MAPK) and adenosine 5’-monophosphate (AMP)-activated protein kinase (AMPK) signaling pathways, triggering significant changes in the hormones and structures of ovulatory follicles and regulating transcription factors and their downstream genes [11,12,13]. PG plays an important role during ovulation. Prostaglandin E2 (PGE2) induces ovulation-related genes (*Areg*, *Ereg*, *Has2*, and *Tnfaip6*) and intracellular cyclic adenosine monophosphate (cAMP) levels. These genes and signaling pathways promote meiosis, cumulus expansion, oocyte maturation, and follicle rupture during ovulation [14,15,16]. The production of steroid hormones in the follicle also plays a crucial role in ovulation. Cytochrome P45017A1 (CYP17A1) has a regulatory effect on steroid synthesis. In the ovary, CYP17A1 is mainly expressed in theca cells, followed by granulosa cells. Low levels of CYP17A1 are expressed in granulosa cells, and the conversion of progesterone into androgen and estrogen is severely restricted, resulting in the synthesis of progesterone [17]. Pituitary gonadotropin (GnRH), FSH, and LH play important regulatory roles in ovarian steroidogenesis [2] and follicle development, while gonadotropin secretion is negatively affected by steroids and positive feedback regulation [18]. 

Sodium salicylate (SS), a nonsteroidal anti-inflammatory drug (NSAID), may exert anti-inflammatory effects by inhibiting the production of PG and the activation of leukocytes [19]. However, there have been conflicting results showing that sodium salicylate may interfere with the induction of PG rate-limiting enzymes (cyclooxygenase 2, COX2). Both the inhibition of COX2 [20,21] and stimulation of COX2 were observed [22,23] in response to sodium salicylate. Furthermore, high levels of arachidonic acid indicate that sodium salicylate inhibits COX2 to reduce prostaglandin formation [24,25]. Studies have shown that AMPK can be directly activated and play an important role in regulating the inflammatory response, including inducing apoptosis, inhibiting cell proliferation and signal transducer and activator of transcription-3 (STAT3) activity, and promoting the secretion of the proinflammatory factors tumor necrosis factor-α (TNF-α) and interleukin-1β (IL-1β) [26,27]. However, current research on low-dose sodium salicylate in ovulation is still unclear and needs to be further examined.

In this study, mice were treated with different doses of sodium salicylate. Low-dose (1.06 mg/kg) sodium salicylate could increase the levels of ovulation hormones and inflammation and simultaneously activate the p38 signaling pathway to promote ovulation. This study not only enriches the selection of superovulation drugs and reduces the use of hormone drugs but also provides a reference for livestock breeding.

## 2. Results

### 2.1. Low Doses of Sodium Salicylate Promoted Ovulation

**Figure 1:**
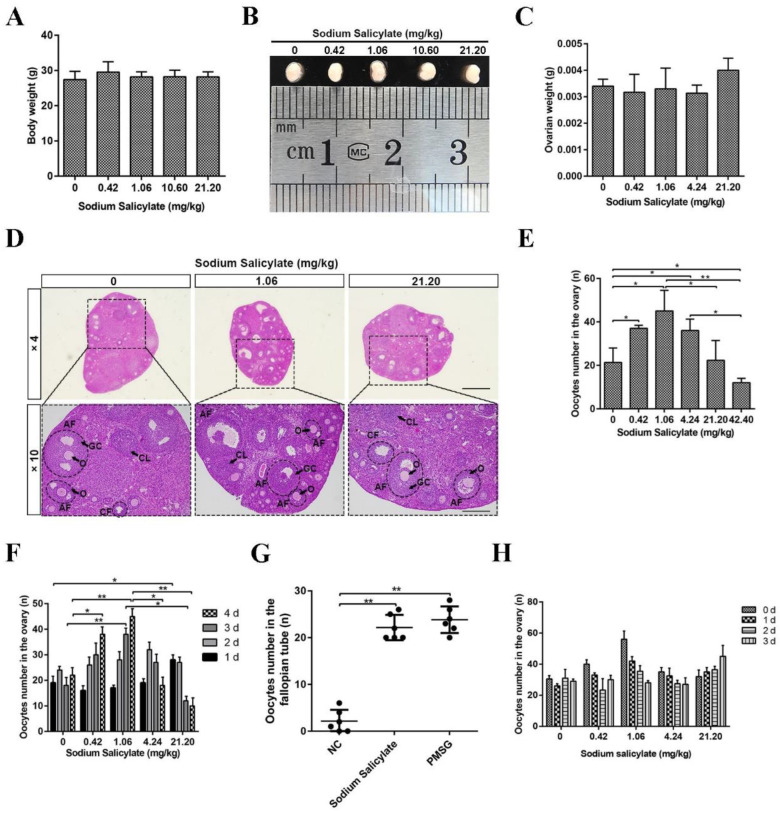
Effects of sodium salicylate on ovulation in mice. (**A**) There was no difference in body weights among the groups treated with various concentrations of sodium salicylate (*n* = 6). (**B**) Effects of different concentrations of sodium salicylate on mouse ovary size. (**C**) Different concentrations of sodium salicylate had no effect on the weight of ovarian tissue (*n* = 6). (**D**) In sections of ovaries treated with different concentrations of sodium salicylate, HE staining showed differences in the numbers of follicles at all levels. (**E**) Effects of different concentrations of sodium salicylate on GV oocyte numbers in ovarian tissue (*n* = 6). (**F**) Relationship between GV oocyte numbers in mouse ovaries and the time of sodium salicylate treatment (*n* = 6). (**G**) Ovulation number in the fallopian tube in response to sodium salicylate (1.06 mg/kg and 21.20 mg/kg) and PMSG (5 IU) treatment (*n* = 6). (**H**) The number of ovulation (GV) changes with the number of days of withdrawal in salicylate-treated mice (*n* = 6). Antral follicles (AFs), cystic follicles (CFs), corpus luteum (CL), granulosa cells (GCs), theca cells (TCs), and oocytes (O). * *p* < 0.05, ** *p* < 0.01. Scale bars, 0.5 mm.

To examine the effect of sodium salicylate on ovulation in this study, mice were intraperitoneally injected with different concentrations of sodium salicylate for four consecutive days. We found that sodium salicylate did not significantly affect body weight (Figure 1A), size (Figure 1B), or ovary weight (Figure 1C). HE staining of ovarian tissue showed that the structure of the ovary was changed in mice treated with sodium salicylate (Figure 1D). Subsequently, the number of oocytes in the ovary was counted, and we confirmed that GV and M2 oocyte numbers were increased by low concentrations of sodium salicylate; 1.06 mg/kg sodium salicylate had the optimal effect on ovulation (Figure 1E,G). However, 42.20 mg/kg sodium salicylate had an inhibitory effect on GV oocyte numbers in the ovary (Figure 1E), and 21.20 mg/kg sodium salicylate did not affect GV oocyte numbers (Figure 1E). Next, we studied the role of sodium salicylate concentrations less than 21.20 mg/kg in promoting ovulation. Within four consecutive days of treatment, the GV oocyte numbers of mice treated with 1.06 mg/kg sodium salicylate gradually increased with time, while those in the 21.20 mg/kg sodium salicylate-treated group gradually decreased (Figure 1F). The GV oocyte number in the 21.20 mg/kg sodium salicylate group was significantly higher than that in the other groups on the first day of administration (*p* < 0.05; Figure 1F). In addition, we counted the number of M2 oocytes in the fallopian tubes, and we found that 1.06 mg/kg had the same effect on superovulation as PMSG, and this was significantly higher than that in the control group (Figure 1G). This is consistent with the results of increased oocytes in the ovaries described above. We also analyzed the recovery of ovulation (GV oocyte) after sodium salicylate treatment. It was found that ovulation in the low-dose sodium salicylate treatment group returned to normal on the third day, while that in the high-dose group took more time (Figure 1H). These results showed that low-dose sodium salicylate could promote ovulation, and 1.06 mg/kg sodium salicylate worked best. 

### 2.2. Mouse Oocyte Quality Was Not Affected by Sodium Salicylate

**Figure 2:**
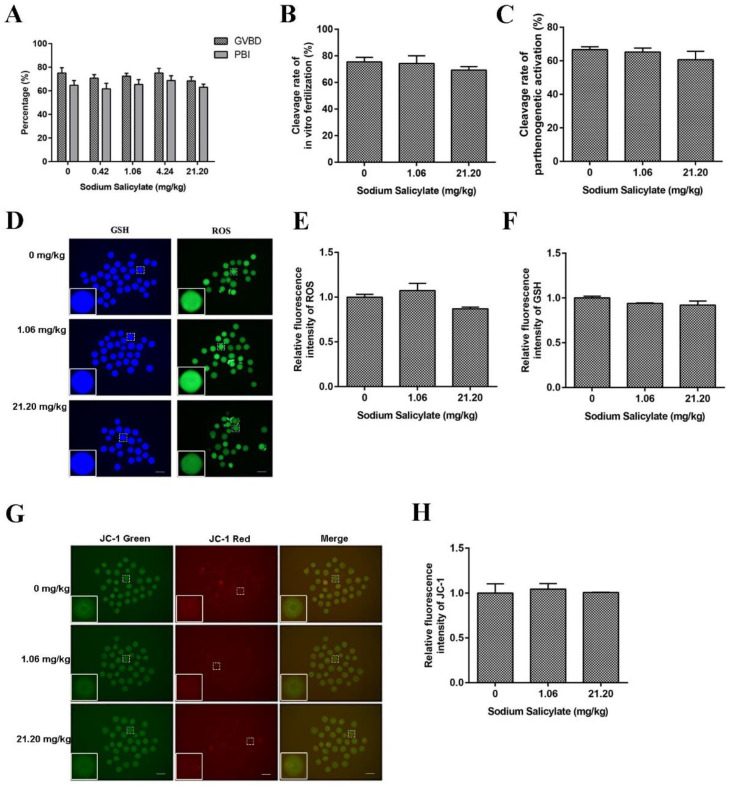
Effects of sodium salicylate on mouse oocytes. (**A**) Oocyte GVBD and PBI rates were not affected by sodium salicylate (*n* = 6). (**B**,**C**) The in vitro fertilization rate (**B**) and parthenogenetic activation of oocytes (**C**) obtained from the ovaries of sodium salicylate-treated mice (*n* = 6) were not affected. (**D**) GSH and ROS fluorescence in oocytes collected from ovaries in sodium salicylate-treated mice (*n* = 3). (**E**,**F**) Fluorescence intensity of ROS (**E**) and GSH (**F**). (**G**) JC-1 fluorescence (red and green channels) was examined to detect the membrane potential of oocytes in the ovaries of mice treated with sodium salicylate (*n* = 3). (**H**) Red/green ratio of JC-1 fluorescence in mouse oocytes (*n* = 3). Scale bars, 100 μm.

To further examine the effect of sodium salicylate on the quality of oocytes, we collected mouse oocytes for maturation cultures. The results showed that there was no effect on the germinal vesicle breakdown (GVBD) rate and first polar body (PB1) rate of oocytes in the control group and different treatment groups (Figure 2A). Neither in vitro fertilization (Figure 2B) nor parthenogenetic activation of oocytes (Figure 2C) showed statistically obvious dissimilarities in cleavage rates. Changes in ROS, GSH, and JC-1 levels are important indicators that reflect the normal development of oocytes. ROS and GSH staining of mouse oocytes showed that the levels of ROS (Figure 2D,E) and GSH (Figure 2D,F) in mouse oocytes were not remarkably different between the sodium salicylate-treated group and the control group. JC-1 staining of oocytes showed no significant differences in the mitochondrial membrane potential of oocytes (Figure 2G,H). These results indicated that low-dose sodium salicylate did not affect oocyte quality. 

### 2.3. Sodium Salicylate Affected the Levels of Hormones and Leukocytes in Mice

**Figure 3:**
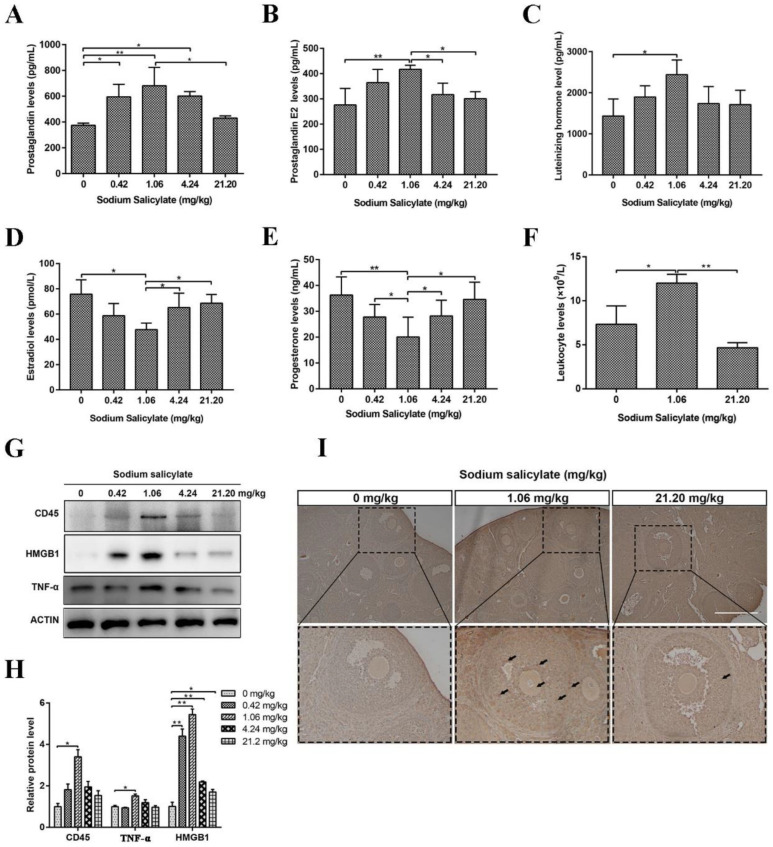
Effects of sodium salicylate on hormones and inflammation in mice. (**A**–**E**) Prostaglandin (**A**), PGE2 (**B**), luteinizing hormone (**C**), estradiol (**D**), and progesterone (**E**) levels in the blood of mice (*n* = 3) treated with various concentrations of sodium salicylate. (**F**,**G**) Protein expression of HMGB1 and TNF-α in mouse ovaries in the different treatment groups. (**H**) Leukocyte levels in the blood of mice treated with different concentrations of sodium salicylate (*n* = 3). (**I**) CD45 (brown) immunohistochemistry of ovarian tissue sections (arrows). Scale bars, 300 μm. * *p* < 0.05, ** *p* < 0.01.

After measuring the hormone levels in the blood of mice, we found that the levels of prostaglandins (PG; Figure 3A), prostaglandin E2 (PGE2; Figure 3B), and luteinizing hormone (LH; Figure 3C) were markedly increased in the 1.06 mg/kg treatment group and that the levels of estradiol (Figure 3D) and progesterone (P4; Figure 3E) were significantly decreased. There were no marked changes in the 21.20 mg/kg sodium salicylate group. This result demonstrated that 1.06 mg/kg sodium salicylate might promote ovulation in mice by changing hormone levels. Tumor necrosis factor-α (TNF-α) and high mobility group box B1 (HMGB1) can reflect the level of inflammation [22]. We found that the protein levels of CD45, TNF-α, and HMGB1 were increased in the ovaries of mice in the 1.06 mg/kg sodium salicylate group (Figure 3F,G). Statistical analysis of leukocytes in the blood showed that 1.06 mg/kg sodium salicylate could increase the level of leukocytes, and this number was significantly higher than that in the 21.20 mg/kg treatment group (*p* < 0.05; Figure 3H). Through CD45 immunohistochemistry of ovarian tissue sections, we found that white blood cells infiltrated the ovary and indeed found that inflammation was strengthened (Figure 3I). These results indicate that 1.06 mg/kg sodium salicylate might promote ovulation by changing hormone levels.

### 2.4. Sodium Salicylate Activated P38 Signaling Pathway and the Key Protein CYP17A1

**Figure 4:**
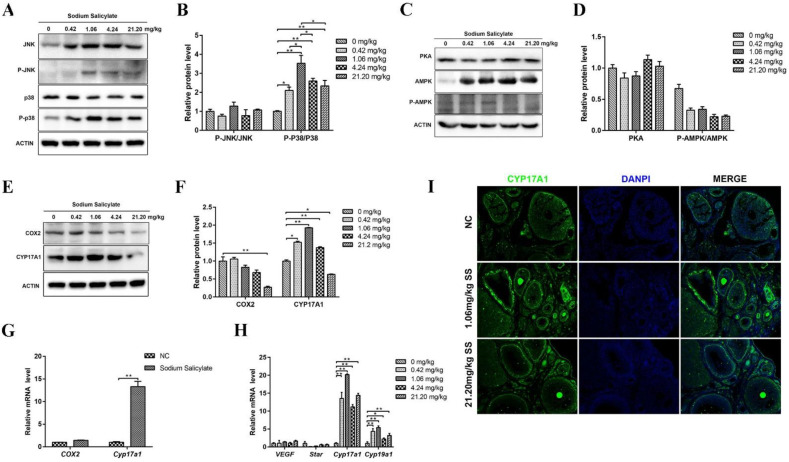
Effects of sodium salicylate on related pathways. (**A**,**B**) Changes in JNK, P-JNK, p38, and P-p38 protein levels in the sodium salicylate (SS)-treated group. (**C**,**D**) Expression of PKA and AMPK pathway-related proteins in the ovaries of mice treated with sodium salicylate (SS) treatment. (**E**–**G**) The protein (**E**,**F**) and mRNA levels (**G**) of COX2 and CYP17A1 were measured in the ovaries of mice in the different treatment groups. (**H**) mRNA expression of *VEGF*, *Star*, *Cyp17a1*, and *Cyp19a1*, which regulate ovulation, in the granulosa cells of mice treated with sodium salicylate. (**I**) Immunofluorescence results of CYP17A1 in ovarian tissue sections. Scale bars, 350 μm. * *p* < 0.05, ** *p* < 0.01.

Studies have shown that the protein kinase A (PKA), protein kinase C (PKC), phosphatidylinositol 3-kinase (PI3K), tyrosine kinase, and the downstream mitogen-activated protein kinase (MAPK) signaling pathways are involved in the development of oocytes in the ovary. There were no changes in total p38 protein levels but a significant increase in phosphorylated p38 (P-p38) (*p* < 0.05; Figure 4A,B). c-Jun N-terminal kinase (JNK) is an important branch of the MAPK pathway that plays an important role in various physiological processes, such as the cell cycle and reproduction [25]. The results showed that the protein levels of total and phosphorylated JNK (P-JNK) were markedly increased (*p* < 0.05; Figure 4A,B), but P-JNK/JNK was not significant changed and affected. There were no significant differences in PKA and adenosine 5’-monophosphate (AMP)-activated protein kinase (AMPK) protein levels in the ovaries in the treatment groups (Figure 4C,D). These data indicate that 1.06 mg/kg sodium salicylate can promote ovulation by activating the p38 signaling pathways.

To further examine the effect of sodium salicylate on ovulation regulators, changes in leukocytes in mice were examined. Cytochrome P45017a1 (CYP17A1) has a regulatory effect on steroid synthesis and indirectly promotes sex hormone production [28]. We found that the protein and mRNA level of CYP17A1 in ovarian tissue was significantly increased (Figure 4E–I). The mRNA level of *Cyp19a1* was also markedly increased in the granulosa cells of mice treated with sodium salicylate (Figure 4H) and in isolated granulosa cells (Figure 4E) after sodium salicylate treatment. COX2 protein levels in ovaries (Figure 4E,F) and mRNA levels in granulosa cells (Figure 4G) showed no significant changes in the 1.06 mg/kg sodium salicylate group. However, COX2 was significantly decreased (*p* < 0.05) in mice treated with 21.20 mg/kg sodium salicylate. Therefore, we hypothesize that 1.06 mg/kg sodium salicylate may regulate steroid hormones to promote ovulation by changing the level of CYP17A1 in the ovary.

### 2.5. Abiraterone Acetate Inhibited the Effect of Sodium Salicylate on Excretion

**Figure 5:**
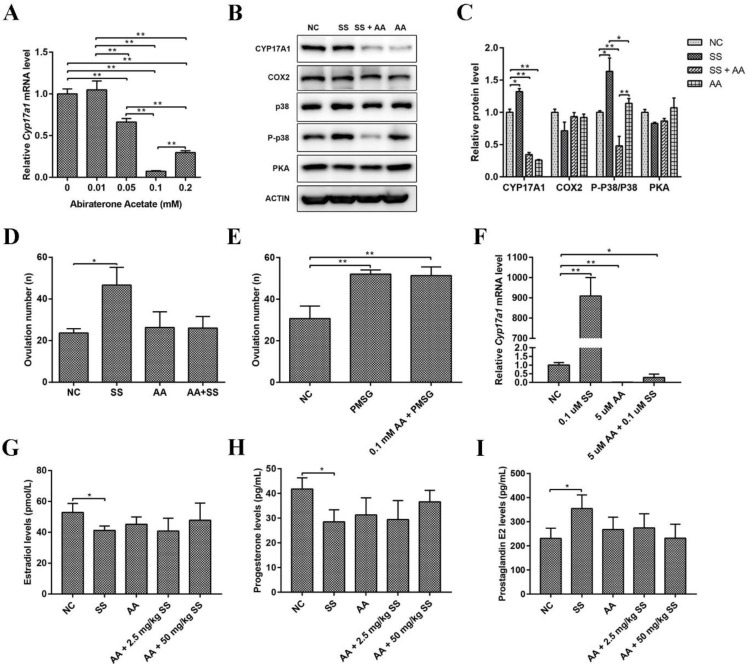
Effects of CYP17A1 inhibition on sodium salicylate. (**A**) Inhibitory effects on ovarian *Cyp17a1* mRNA levels after the intraperitoneal injection of abiraterone acetate (AA). (**B**,**C**) Impact of abiraterone acetate on CYP17A1, COX2, P-p38, and PKA protein levels in the ovary. (**D**) Abiraterone acetate inhibited the superovulation effect of sodium salicylate (SS; *n* = 6). (**E**) Abiraterone acetate did not affect the superovulation effect of PMSG (*n* = 6). (**F**) The mRNA expression of *Cyp17a1* was inhibited by the addition of abiraterone acetate to granulosa cells in vitro. (**G**–**I**) Changes in estradiol (**G**), progesterone (**H**), and prostaglandin E2 (**I**) in the blood of mice after the intraperitoneal injection of abiraterone acetate (*n* = 3). * *p* < 0.05, ** *p* < 0.01.

To further verify our hypothesis, mice were injected with abiraterone acetate (a CYP17A1 inhibitor, AA), followed by sodium salicylate treatment (1.06 mg/kg) 30 min later. The best concentration of AA was subsequently determined to verify the effect of sodium salicylate. As shown in Figure 5A, 0.0392 mg/kg (1 mM) AA was the optimal concentration to inhibit the expression of *Cyp17a1*. Ovulation-related protein levels in the ovary were examined, and we found that the protein level of CYP17A1 was markedly inhibited by abiraterone acetate (AA) in the sodium salicylate treatment group and that the P-p38/p38 protein level was significantly reduced, while the protein levels of COX2 and PKA were not obviously changed (Figure 5B,C). Moreover, after AA treatment, superovulation did not occur and returned to normal levels in response to 1.06 mg/kg sodium salicylate (Figure 5D). Thus, these results indicated that sodium salicylate increased the number of ovulations in mice by promoting the expression of CYP17A1. Next, we examined whether PMSG was also regulated by CYP17A1 or affected superovulation induced by PMSG (5 IU) in mice. PMSG can promote ovulation after inhibition mediated by abiraterone acetate (Figure 5E). The production of steroid hormones in follicles is crucial for successful ovulation. Granular cells rapidly accumulate cholesterol-containing lipid droplets, providing the raw material for the synthesis of steroid hormone precursors. Moreover, CYP17A1 is also expressed in granulosa cells in the ovary [10], and this investigation was performed on granulosa cells. Abiraterone acetate inhibited the increase in the expression of Cyp17a1 induced by sodium salicylate in granulosa cells (Figure 5F). AA restored hormone (Estradiol, P4 and PGE2) changes caused by SS (Figure 5G–I). These results demonstrate that 1.06 mg/kg sodium salicylate can promote ovulation by regulating the expression of CYP17A1.

### 2.6. Superovulation Was Induced by Sodium Salicylate and FSH/PMSG

**Figure 6:**
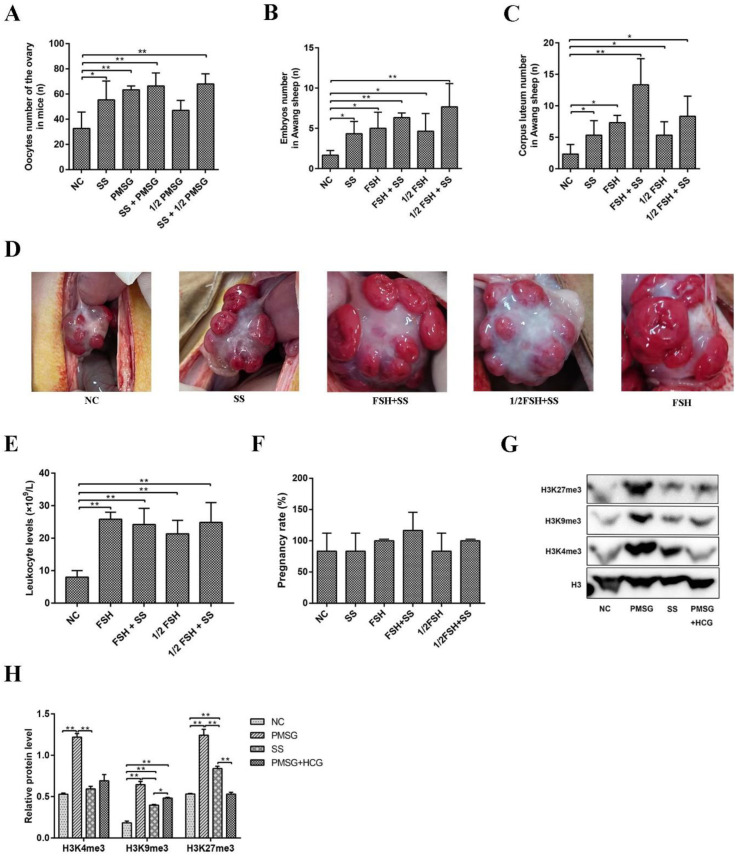
Effects of sodium salicylate and FSH/PMSG on superovulation. (**A**) Ovulation in mice was affected by the combination of sodium salicylate (SS) and PMSG (*n* = 4). (**B**,**C**) Influence of the combination of sodium salicylate (SS) and FSH on the number of embryos (**B**) and corpora luteum (**C**) in Awang sheep (*n* = 4). (**D**) Sodium salicylate (SS) and FSH increased the level of leukocytes in the blood of Awang sheep in the different treatment groups (*n* = 4). (**E**) Different treatments affected the appearance of the ovarian corpus luteum in Awang sheep (*n* = 4). (**F**) Pregnancy rate of Awang sheep. Two embryos were transferred to each sheep (*n* = 6). (**G**,**H**) SS induced weaker histone methylation levels than those with PMSG. * *p* < 0.05, ** *p* < 0.01.

Considering the effect of sodium salicylate on superovulation, we used different combinations of sodium salicylate and FSH/PMSG. Sodium salicylate and FSH/PMSG increased the ovulation number in mice (*p* < 0.05; Figure 6A and Figure 1H). The combination of sodium salicylate + 1/2 PMSG increased the amount of superovulation in mice compared with 1/2 PMSG treatment group (Figure 6A). Similarly, sodium salicylate and FSH significantly increased the number of embryos (Figure 6B) and the number of corpora lutea (Figure 6C) in Awang sheep undergoing superovulation. The combination of sodium salicylate and 1/2 FSH induced superovulation similar to that with FSH (Figure 6B,C). Ovarian ovulation was then observed via abdominal surgery. After superovulation was induced in Awang sheep by 1/2 FSH + sodium salicylate or FSH + sodium salicylate, the number of corpora lutea on the ovarian surface increased significantly (Figure 6C,D). The use of FSH, sodium salicylate + FSH, 1/2FSH, and sodium salicylate + 1/2FSH significantly increased leukocyte levels during superovulation (Figure 6E). There was no difference in the number of lambs born after embryo transfer in Awang sheep with SS superovulation (Figure 6F). Compared to that with PMSG stimulation, sodium salicylate stimulation significantly reduced the histone methylation level (Figure 6G,H). Therefore, sodium salicylate may play a role in superovulation induced by FSH/PMSG and can be used as an additive for FSH/PMSG superovulation drugs, which can greatly reduce the use of hormone drugs.

## 3. Discussion

This study showed that low-dose sodium salicylate (<21.20 mg/kg) could increase the levels of ovulation hormones and inflammation by promoting the expression of CYP17A1 and that sodium salicylate activated the p38 signaling pathway to promote ovulation. We used sodium salicylate to verify superovulation in mice and Awang sheep and found that sodium salicylate had the same effect as the commonly used superovulation drug pregnant horse serum gonadotropin (PMSG)/follicle-stimulating hormone (FSH) and could also be used as an additive for FSH/PMSG superovulation drugs. Sodium salicylate not only expanded the options for superovulation but also greatly replaced or reduced the use of hormones in the breeding of livestock via superovulation.

This study showed that 1.06 mg/kg sodium salicylate markedly induced ovulation, but 21.20 mg/kg sodium salicylate had no effect on ovulation in mice. Interestingly, treatment with 1.06 mg/kg sodium salicylate for four consecutive days or 21.20 mg/kg sodium salicylate for 1 d induced superovulation in mice. The rate of oocyte maturation in vitro and the cleavage rate of in vitro fertilization and parthenogenesis are important indicators of oocyte quality. The use of this drug did not change mouse body weight or ovary weight. Oxidative stress is one of the important factors affecting the development of oocytes, and excessive reactive oxygen species (ROS) levels can inhibit the development of oocytes [18]. Glutathione (GSH) also plays an important role in maintaining oocyte quality and is an essential antioxidant in oocytes [19]. Decreased GSH levels can lead to DNA damage and increases in ROS levels in oocytes [20]. Oocytes can mature and develop normally, and ROS, GSH, and mitochondrial membrane potential are not disturbed. Therefore, follow-up studies should be carried out.

Since hormones and inflammation affect ovulation, whether sodium salicylate plays a role in promoting ovulation by regulating hormone and inflammatory responses warrants investigation.

Ovulation is regulated by various hormones, including luteinizing hormone (LH) [2], prostaglandins (PG), estradiol, and progesterone. Ovulation events are triggered by LH surges [10]. The changes in LH, PG, E2, and progesterone in blood were consistent with the hormone levels required for ovulation in mice. The LH surge triggers ovulation events and initiates the signaling network. These changes lead to follicle rupture and oocyte release. In response to sodium salicylate, the level of LH in mice increased dramatically and promoted oocyte maturation and ovulation. PG also plays an important role during ovulation, and prostaglandin E2 (PGE2) induces the expression of ovulation-related genes, such as *Areg*, *Ereg*, *Has2*, and *Tnfaip6*, and affects intracellular cyclic adenosine monophosphate (cAMP) levels, influencing meiosis, cumulus expansion, and follicular rupture [16]. When the level of PGE2 increases with the LH surge, gonadotropins promote the expansion of cumulus cells and the expression of proteases related to follicular wall rupture [14,15,29,30], providing the conditions for successful ovulation to ensure that there are sufficient expanded cumulus cells. The LH surge that regulates prostaglandin metabolism is widely recognized as a necessary and rate-limiting step during ovulation in all mammalian species. Our results showed that sodium salicylate increased the levels of PG, PGE2, and LH in the blood and promoted ovulation. Progesterone is a key regulator of reproductive events, including ovulation and luteinization [12,31,32,33,34]. Furthermore, progesterone has been shown to be involved in inflammatory responses in various tissues and can act as a pro- or anti-inflammatory modulator [35,36,37,38]. Decreased levels of estradiol and progesterone hormones promote ovulation, which is consistent with hormonal changes during normal PMSG stimulation and sodium salicylate treatment. 

Inflammation is an important condition for ovulation, and inhibiting or activating inflammation can regulate ovulation in animals. For example, the levels of the cytokine interleukin-8 (IL-8) are low in the anovulatory ovaries of cattle, but the levels of IL-8 increase rapidly after the LH surge in granulosa and theca cells [39]. Inhibiting IL-8 or neutrophils can reduce the number of ovulations in rabbits [40]. Tumor necrosis factor-α (TNF-α) is expressed in all leukocytes as an early proinflammatory factor [41]. High mobility group box B1 (HMGB1) plays an important role in the inflammatory process [42]. Sodium salicylate increased the expression of TNF-α and HMGB1 in the ovary. It also increased the number of leukocytes in the blood. Ovulation was promoted by enhancing the level of inflammation in the ovary. This finding is consistent with studies reporting that inflammation promotes ovulation. Therefore, sodium salicylate may promote ovulation by altering inflammation and hormone levels.

The regulatory effects on steroids, oocyte maturation, and final oocyte release do not require inflammation during ovulation [10]. Steroids can selectively modulate the secretion of follicle stimulating hormone (FSH) and LH [43]. Successful ovulation requires the production of intrafollicular steroids. Steroids are also powerful vascular modulators in the reproductive tract. It has been reported that steroid hormones can induce the synthesis of sex hormones to promote leukocyte secretion [44,45,46,47,48,49,50]. CYP17A1 is a key steroid hormone synthesis monooxygenase for steroid-to-androgen conversion [28]. Androgen plays an important role in normal follicular maturation and female fertility. Androgen mainly plays a role in preantral and early antral follicles and regulates the activity of FSH in granulosa cells and the number of leukocytes in the blood through transcriptional regulation of the androgen receptor (AR) [51]. Sodium salicylate promoted the expression of CYP17A1 and increased the level of androgen receptor, which was consistent with the increase in the level of white blood cells in the blood and the promotion of ovulation. Estrogen and progesterone also play regulatory roles in ovulation. Their levels changed correspondingly during the 1.06 mg/kg sodium salicylate-mediated promotion of ovulation. Abiraterone acetate (AA) eliminated sodium salicylate-induced superovulation by inhibiting the protein expression of CYP17A1 in the ovary. Thus, we concluded that 1.06 mg/kg sodium salicylate regulates steroid hormone/leukocyte hormones and controls ovulation by regulating the expression of CYP17A1.

Under a complicated signaling network in the cell, the LH surge in preovulatory follicles exerts ovulatory effects, affecting the protein kinase A (PKA), protein kinase C (PKC), phosphatidylinositol 3-kinase (PI3K), and mitogen-activated protein kinases (MAPK) pathways. This study indicates that sodium salicylate mainly acts through the p38 signaling pathway during ovulation induction, which is inconsistent with the general presumption that the intracellular PKA signaling pathway is the main pathway in preovulatory follicles mediated by luteinizing hormone (LH)/human chorionic gonadotropin (hCG). Consistent with these results, the p38 signaling pathway can activate the MAPK signaling network in the follicle without upstream PKA proteins [22,23]. Our results demonstrate that sodium salicylate activates the p38 pathway to promote ovulation.

In this study, mice that had not been treated with superovulation hormones were selected as research subjects. In addition, Awang sheep living in Tibet without manual intervention were selected as subjects to verify superovulation to circumvent interference from other superovulation drugs. At present, superovulation drugs that are prevalent in animal reproduction and breeding include FSH and pregnant horse serum gonadotropin (PMSG). The use of these drugs often leads to disturbances in hormone levels, causing inflammation and changes in histone methylation levels. Moreover, hormone drugs are expensive, and transportation and storage conditions are harsh, which greatly increases the cost of breeding, while sodium salicylate has the advantages of a low price, room temperature storage, and convenient availability. Sodium salicylate has the same effect on superovulation in mice as FSH/PMSG. The combination of sodium salicylate and 1/2 PMSG could also achieve the same effect and compensate for the effect of PMSG. In Awang sheep, sodium salicylate also had the same effect as FSH on superovulation, and it could achieve the same effect when combined with 1/2 FSH. The number of embryos and corpus lutea increased significantly, and the individuals were plump. Therefore, sodium salicylate may have the same effect as FSH/PMSG on superovulation and can be used as an additive or a substitute for FSH/PMSG, which can greatly reduce the dose and frequency of hormone drugs in production.

At present, the effects of sodium salicylate as an anti-inflammatory drug in the study are consistent with the results of high-dose sodium salicylate (42.40 mg/kg)-mediated inhibition of COX2. However, low-dose sodium salicylate did not affect the expression of COX2 and induced superovulation by promoting the expression of CYP17A1. CYP17A1 increased LH and steroid hormone levels, leading to an increase in proinflammatory factors and leukocyte levels.

**Figure 7:**
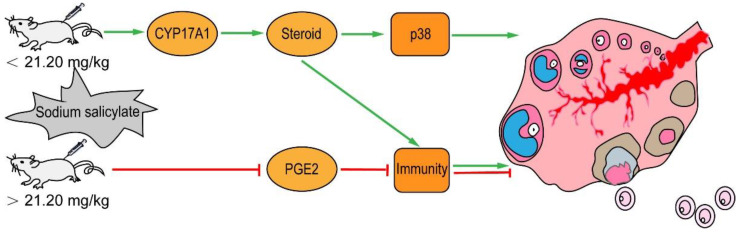
Low-dose sodium salicylate regulates the increase in endosterol and PGE2 through CYP17A1 and ultimately activates the p38 signaling pathway to promote ovulation. Ovulation, shown in green, is promoted by low-dose sodium salicylate injection. Ovulation, shown in red, is inhibited by high-dose sodium salicylate injection.

In conclusion, 1.06 mg/kg sodium salicylate promotes ovulation by modulating the expression of CYP17A1 and by regulating endosterol to increase the levels of ovulation hormones and inflammation and activate the p38 signaling pathway (Figure 7). Therefore, sodium salicylate can be used as a superovulation drug or additive to reduce the cost of breeding and help in the rapid development of the cultivation industry.

## 4. Materials and Methods

### 4.1. Chemicals

Sodium salicylate (SS), M2, DMEM/F-12 medium, Mineral oil, and Milrinone were obtained from Sigma-Aldrich. Trizol, MEM, and DMEM were obtained from Invitrogen. Primer synthesis services were obtained from Sangon Bioengineering (Shanghai, China). The bicinchoninic acid (BCA) was obtained from Takara (Japan). Glutamine, non-essential amino acids, fetal bovine serum, and trypsin digestive enzymes were obtained from Gibco. Pregnant Mare Serum Gonadotropin (PMSG), Luteinizing Hormone, and Follicle-stimulating hormone (FSH) were obtained from NingBo SanSheng biolocical technology CO., LTD. Abiraterone acetate (CB7630) was purchased from MedChem Express. 

### 4.2. Animals and Treatment

Adult female ICR mice (4–6-weeks-old, weighing 20–25 g) were purchased from the animal center of the Chengdu Dossy experimental Animal CO., LTD (Xi`an, China). The laboratory conditions for animal maintenance were as follows: 22 ℃ ± 2 ℃, 55% ± 5% relative humidity with a 12 h light/dark cycle, and feeding ad libitum. Animal welfare was implemented according to the guidelines of the care and use of Laboratory Animals in the Northwest A&F University. For optimal drug concentration screening, ICR female mice were randomly divided into 5 groups with 6 mice in each group, and 0.1 mL sodium salicylate (0 mg/kg, 0.424 mg/kg, 1.06 mg/kg, 4.24 mg/kg, and 21.20 mg/kg) was intraperitoneally injected for 4 consecutive days. In the inhibition test, mice were injected intraperitoneally with 0.1 mL abiraterone acetate (CYP17A1 inhibitor, AA; 0.1 mL 5% benzyl alcohol and 95% safflower oil solution; 0.0039 mg/kg, 0.0186 mg/kg, 0.0392 mg/kg and 0.0784 mg/kg) for 30 min and then treated with sodium salicylate (1.06 mg/kg) for 4 consecutive days. The treatment methods of AA and PMSG (5 IU) were the same as above, only for one day. Mice in the normal group were classified as being in the estrus/metestrus stage of the natural estrous cycle by observing vaginal congestion by eye [52], as well as by evaluating the results of vaginal smears collected under a dissecting microscope.

The Awang sheep were selected from the Changdu Breeding Plant in Tibet, and they were 50–60 kg young ewes aged 2 to 3 years and were grazing and raised year round. The method of combining self-fertilization and artificial fertilization was adopted for sheep fertilization to ensure full fertilization. The embryos were recovered from the uterine horn via surgery. Sodium salicylate and PMSG (mouse superovulation drug)/FSH (ovine superovulation drug) were compared in the superovulation test, which included intraperitoneal injection of physiological saline (NC), sodium salicylate (SS; 1.06 mg/kg), PMSG (5 IU)/FSH (500 IU), 1/2 PMSG/FSH, 1/2 PMSG/FSH + sodium salicylate (SS), and PMSG/FSH + sodium salicylate (SS). After treatments, ovaries, oocytes, and granulosa cells were collected for detection and statistical analysis. Animal care was approved by the Animal Care and Use Committee of the Northwest University of Agriculture and Forestry Institution and conformed to the national laws and regulations of relevant departments.

### 4.3. Collection of Oocytes and Embryo

#### 4.3.1. Collection of Mouse Oocytes

The development of oocytes in the ovary lays the foundation for superovulation. The ovaries were collected 44 h after treatment in different groups. The ovaries on both sides were put into MEM culture medium. All oocytes in the whole ovary were separated from the follicles with sterile needles, and the number of oocytes was counted after digestion with 0.3% hyaluronidase (sigma-Aldrich). Germinal vesicle (GV)-stage oocytes to be matured were collected and counted under the stereomicroscope.

The superovulation of mice was analyzed, and the mature oocytes in fallopian tubes were collected and counted. After the above treatment, the mice were injected with 5 IU HCG (Ningbo Sansheng, China), and mature oocytes were collected within 17–20 h. Mouse cumulus-oocytes were collected from fallopian tubes and treated with 0.3% hyaluronidase (sigma-Aldrich) to count oocytes after digestion.

#### 4.3.2. Collection of Embryos from Awang Sheep

On the 6th day of breeding, the embryos were washed with the embryo flushing fluid, collected from the outlet on the other side, and then screened and counted under the microscope.

### 4.4. In Vitro Fertilization

Mature mouse oocytes were collected using the above methods. The cumulus-oocyte mass was depleted of cumulus cells with 0.25% hyaluronidase and then fertilized with freshly diluted sperm in capacitative fluid for 5 h. After fertilization, the oocytes were washed in KSOM medium 3 times. The cells were cultured in an incubator at 37 °C, with 5% CO_2_, and saturated humidity, and the cleavage rate was determined.

### 4.5. Parthenogenetic Activation

Through the above methods, mature oocytes are parthenogenetically activated. Mature oocytes were washed once in Hepes-CZB without Ca^2+^, placed in electric shock solution for 2–3 min, and then subjected to a voltage of 200 V, electric shock time of 80 μs, and electric shock 3 times and then rinsed with Hepes-CZB and transferred to KMSO for 6 h; finally, the activated embryos were cultured and observed in the incubator.

### 4.6. Detection of ROS Levels in Oocytes

Reactive oxygen species (ROS) levels in oocytes were detected with a Reactive Oxygen Species Assay Kit (Beyotime, S0033S). The oocytes were transferred to pre-warmed DCFH-DA working solution, incubated at 37 °C for 20 min, and then washed three times with PBS. The level of ROS in oocytes was observed with a fluorescence microscope. Next, we analyzed the average fluorescence intensity with Image J. Experiments were repeated three times, and 150 oocytes were counted.

### 4.7. Detection of GSH Level in Oocyte

Glutathione (GSH) levels in oocytes were detected with a glutathione kit (Beyotime, S0052). The oocytes were transferred to pre-warmed Thiol Tracker^TM^ Violet working solution, incubated at 37 °C for 30 min, and washed with PBS. GSH levels were evaluated under a fluorescent microscope. Image J was used to analyze the average fluorescence intensity of GSH in oocytes. Experiments were repeated three times, and each time, we counted 50 oocytes.

### 4.8. Mitochondrial Membrane Potential Determination in Oocytes

The mitochondrial membrane potential assay kit (Beyotime, S2006) with JC-1 was used to detect the oocyte membrane potential. Oocytes were washed three times in pre-warmed PBS, then transferred to JC-1 staining working solution, and incubated at 37 °C for 20 min. Subsequently, we analyzed fluorescence intensity with Image J and calculated the membrane potential in oocytes. Experiments were repeated three times, and 150 oocytes were counted.

### 4.9. Cell Culture and Treatment

The granulosa cells (GC) were harvested from ICR dam ovaries. Mouse ovaries were harvested and individually transferred into preheated PBS. GCs were collected via follicle puncture under a microscope. The cells were cultured in DMEM/F-12 medium (Sigma, D0697) supplemented with 10% fetal bovine serum (Gibco, 10270) and 100 units/mL penicillin plus 100 μg/mL streptomycin (Gibco, 15140) for 4 d at 37 °C with 5% CO_2_. GCs were maintained in F12 medium (Gibco, America) containing 5% fetal bovine serum (Sigma-Aldrich) and incubated at 37 ℃ with 5% CO_2_. To inhibit CYP17A1 levels, cells were treated with abiraterone acetate (AA), and then, sodium salicylate (SS) was administered for 24 h to detect the effect. Cell samples were collected to explore the mechanism of sodium salicylate. 

### 4.10. RNA Isolation, Reverse Transcription, and Quantitative PCR

Total RNA was extracted using the RNeasy Mini kit (QIAGEN) according to the manufacturer’s instructions, and each RNA sample was divided into three samples and reverse transcribed according to the manufacturer’s instructions. Quantitative PCR (qPCR) analysis was performed using Power SYBR Green PCR Master Mix (Applied Biosystems, Life Technologies) and the Cfx Connect F1902 Real-Time PCR System. Relative mRNA levels were normalized to the level of *β-actin* mRNA (internal control). The primers are listed in Table 1.

### 4.11. Western Blot Analysis

The protein samples were added to RIPA Lysis Buffer and centrifuged at 1400 rpm for 5 min. The protein concentration was detected using a BCA Kit. The samples were subjected to protein electrophoresis, transfer, and finally to antibody (Table 2) incubation. The results were detected using an ECL method. Finally, Image J software was used for data analysis.

### 4.12. Histological Analysis

The ovarian tissue was fixed in fixative solution for 10–12 h, de-fatted in xylene, dehydrated through a series of increasing concentrations of ethanol, and finally embedded in paraffin. Then, we used a microtome to cut tissue samples and mounted them onto glass slides. The tissue sections were dewaxed with xylene, rehydrated in a series of ethanol with decreasing concentrations, stained with hematoxylin for 1 min, differentiated with 5% glacial acetic acid for 30 s, and treated with distilled water for 15 min, followed by eosin staining for 20 s. Finally, observation was performed with a stereomicroscope.

### 4.13. Immunofluorescence Analysis

Immunofluorescence (IF) assays were used to evaluate the expression of CYP17A1 in ovarian tissues. Sections were deparaffinized, rehydrated, permeabilized, exposed to the CYP17A1 primary antibody, and incubated overnight at 4 ℃. Next, sections were incubated with corresponding secondary antibodies. Nuclei were counterstained with DAPI. Finally, images were obtained with a fluorescence microscope.

### 4.14. Immunohistochemistry

For immunohistochemistry (IHC), processed paraffin ovary sections were sectioned as above and stained with primary antibodies against CD45 overnight at 4 ℃. Next, they were incubated with the corresponding secondary antibodies and the DAB kit. The sections were finally visualized under a microscope.

### 4.15. Hormone Determination

Samples were collected and the enzyme-linked immunosorbent assay (ELISA) was performed using a 96-well kit according to the kenuodi manufacturer’s instructions. A microplate reader (Rayto, Rt-6100) was used to determine the concentrations of hormones, such as luteinizing hormone (LH; kenuodi, CK-E20343), PG (kenuodi, CK-E21031), PGE2 (kenuodi, CK-E20655), estradiol (kenuodi, CK-E20381), and progesterone (P4; kenuodi, CK-E20376).

### 4.16. Leukocyte Counts

Leukocytes were measured via automatic counting using an automatic blood cell analyzer (URIT-2900PlusVet).

### 4.17. Statistical Analysis

The experiment consisted of at least 3 independent samples and at least 3 replicates. The statistical analysis adopted ANOVA to compare the differences in each group. In the results, *p* < 0.05 and *p* < 0.01 were represented by asterisks, (*) and (**), respectively, indicating statistical significance.

## Figures and Tables

**Table 1:** Primer information.

Gene	Forward Primer (5’→3’)	Reverse Primer (5’→3’)	Product Size, bp
*Cox2* *Cyp17a1* *VEGF* *Star* *Cyp19a1*	CAGCCAGGCAGCAAATCCTT TGGAGGCCACTATCCGAGAA GCACTGGACCCTGGCTTTACT AACGGGGACGAAGTGCTAAG ATCCGGTTTTTAAACGGCTGC	GTCCGGGTACAGTCACACTT GAAGCGCTCAGGCATAAACC TCTCAATCGGACGGCAGTAG CCTCTGCAGGACCTTGATCTC TCTTGCGCTATTTGGCCTGG	99 194 143 168 100
*β-actin*	TCTTTTCCAGCCTTCCTTCTTG	GTTGGCATAGAGGTCTTTACGGA	109

**Table 2:** The antibody information.

Antibody	Species Source	Supplier	Identifier	Dilution	
				WB	IF/IHC
P-AMPK	Rabbit	Abcam	ab133448	1:1000	
CYP17A1	Rabbit	Proteintech	14447-1-AP	1:1000	1:200
COX2	Rabbit	Proteintech	12375-1-AP	1:1000	
PKA	Rabbit	Proteintech	55388-1-AP	1:1000	
TNF-α	Mouse	Proteintech	60291-1-Ig	1:1000	
HMGB1	Rabbit	Proteintech	10829-1-AP	1:1000	
H3K4me3	Rabbit	Cell Signaling Technology	9751	1:1000	
H3K9me3	Rabbit	Cell Signaling Technology	13969	1:1000	
H3K27me3	Rabbit	Cell Signaling Technology	9733	1:1000	
H3	Rabbit	Proteintech	17168-1-AP	1:1000	
AMPK	Mouse	Santa Cruz Biotechnology	sc-74461	1:500	
JNK	Mouse	Cell Signaling Technology	9252	1:1000	
P-JNK	Rabbit	Cell Signaling Technology	4668	1:1000	
p38	Mouse	Santa Cruz Biotechnology	sc-7972	1:500	
P-p38	Mouse	Santa Cruz Biotechnology	sc-166182	1:500	
β-actin	Mouse	CWBIO	CW0096	1:2000	
CD45	Mouse	Santa Cruz Biotechnology	sc-1178	1:500	1:100

## Data Availability

All data generated or analyzed during this study are included in this published article.

## Abbreviations

SS	Sodium salicylate
PMSG	Pregnant horse serum gonadotropin
FSH	Follicle-stimulating hormone
CYP17A1	Cytochrome P45017A1
LH	Luteinizing hormone
HCG	Human chorionic gonadotropin
MAPK	Mitogen-activated protein kinase
AMPK	Adenosine 5’-monophosphate (AMP)-activated protein kinase
PG	Prostaglandin
PGE2	Prostaglandin E2
COX2	Cyclooxygenase 2
CAMP	Cyclic adenosine monophosphate
NSAID	Non-steroidal anti-inflammatory drug
TNF-α	Tumor necrosis factor-α
IL-1β	Interleukin-1β
IL-8	Interleukin-8
AA	Abiraterone acetate
ROS	Reactive oxygen species
GSH	Glutathione
GC	Granulosa cells
GV	Germinal vesicle
GVBD	Germinal vesicle breakdown
PB1	Polar body first
PKA	Protein kinase A
PKC	Protein kinase C
PI3K	Phosphatidylinositol 3-kinase
JNK	c-Jun N-terminal kinase
IF	Immunofluorescence
IHC	Immunohistochemistry
DAPI	4’,6-Diamidino-2-phenylindole
DAB	3,3’-Diaminobenzidine
